# Interleukin-6: an emerging regulator of pathological pain

**DOI:** 10.1186/s12974-016-0607-6

**Published:** 2016-06-07

**Authors:** Ya-Qun Zhou, Zheng Liu, Zhi-Heng Liu, Shu-Ping Chen, Man Li, Allahverdi Shahveranov, Da-Wei Ye, Yu-Ke Tian

**Affiliations:** Department of Anesthesiology and Pain Medicine, Tongji Hospital, Tongji Medical College, Huazhong University of Science and Technology, Wuhan, China; Department of Urology, Tongji Hospital, Tongji Medical College, Huazhong University of Science and Technology, Wuhan, China; Department of Anesthesiology, Shenzhen Second People’s Hospital, Shenzhen, China; Department of Neurobiology and Key Laboratory of Neurological Diseases of Hubei Province, Tongji Medical College, Huazhong University of Science and Technology, Wuhan, China; Cancer Center, Tongji Hospital, Tongji Medical College, Huazhong University of Science and Technology, Wuhan, China

**Keywords:** Interleukin-6, Bone cancer pain, Neuropathic pain, Inflammatory pain

## Abstract

Interleukin-6 is an inflammatory cytokine with wide-ranging biological effects. It has been widely demonstrated that neuroinflammation plays a critical role in the development of pathological pain. Recently, various pathological pain models have shown elevated expression levels of interleukin-6 and its receptor in the spinal cord and dorsal root ganglia. Additionally, the administration of interleukin-6 could cause mechanical allodynia and thermal hyperalgesia, and an intrathecal injection of anti-interleukin-6 neutralizing antibody alleviated these pain-related behaviors. These studies indicated a pivotal role of interleukin-6 in pathological pain. In this review, we summarize the recent progress in understanding the roles and mechanisms of interleukin-6 in mediating pathological pain associated with bone cancer, peripheral nerve injury, spinal cord injury, chemotherapy-induced peripheral neuropathy, complete Freund’s adjuvant injection, and carrageenan injection. Understanding and regulating interleukin-6 could be an interesting lead to novel therapeutic strategies for pathological pain.

## Background

Pathological pain is characterized by a low threshold and an exaggerated response to noxious stimuli, and it can be categorized as cancer pain, neuropathic pain, or inflammatory pain [1, 2]. Although physiological pain is essential for the elimination of damaging stimuli, pathological pain significantly affects the quality of life [3–5]. Currently, pathological pain is thought to be mainly induced by a combination of peripheral drives and central processing [6–9]. Despite growing knowledge of the mechanisms of pathological pain, this type of pain still represents a major challenge in clinical practice and basic science. Cytokines have been reported to participate in the regulation of numerous cellular functions including the inflammatory response and expression of cell surface proteins [10–12]. In addition, we previously reported that several cytokines could potentially serve as targets for the management of bone cancer pain (BCP) [13–19]. Recently, mounting evidence has suggested that one cytokine in particular, interleukin-6 (IL-6), may play a critical role in the development of pathological pain [20–24].

IL-6 is an inflammatory cytokine with wide-ranging biological effects. It was first described as B-stimulatory factor 2, which induces B lymphocytes to produce immunoglobulin [25]. IL-6 exerts its biological effect on target cells by interacting with the non-signaling membrane-bound IL-6 receptor (mIL-6R) [26, 27]. The IL-6 and mIL-6R complex then associates with the signal transducing membrane protein gp130, promoting its dimerization and the subsequent activation of intracellular signaling including the Janus-activated kinase/signal transducer activator of transcription (JAK/STAT), mitogen-activated protein kinase/extracellular signal-regulated kinase (MAPK/ERK), and phosphatidylinositol 3-kinase/protein kinase B (PI3K/Akt) signaling pathways [28–30]. This manner of IL-6 signaling is often referred to as “classical IL-6 signaling.” gp130 is expressed by almost all cells in the body, whereas the mIL-6R has a highly restricted expression profile, and is mainly expressed by hepatocytes, neutrophils, monocytes/macrophages and certain other leukocytes [31, 32]. Only cells expressing mIL-6R can bind and respond to IL-6. Thus, until the discovery of a naturally occurring soluble form of IL-6R (sIL-6R), it was difficult to understand how IL-6 could elicit wide-ranging biological responses by interacting with a limited number of cell types. sIL-6R has been found in various body fluids and has been shown to be generated by two independent mechanisms: limited proteolytic cleavage from mIL-6R and translation from a differentially spliced messenger RNA (mRNA) [33–35]. A complex comprising IL-6 and sIL-6R is also able to bind to gp130 and to initiate intracellular signaling [36, 37]. Through this so-called “trans-signaling” mechanism, IL-6 is capable of stimulating cells that lack endogenous mIL-6R [38]. Additionally, it has been shown that the soluble form of gp130 (sgp130) exclusively inhibits IL-6 responses mediated via the IL-6/sIL-6R complexes (i.e., trans-signaling) and does not affect stimulation via mIL-6R (i.e., classical IL-6 signaling) [39–41]. Therefore, sgp130 can be used as a molecular tool to discriminate between classical signaling and trans-signaling.

Various pathological pain models have shown elevated expression levels of IL-6, IL-6R, and gp130 in the spinal cord and dorsal root ganglia (DRG). Additionally, the administration of IL-6 could cause mechanical allodynia or thermal hyperalgesia, and an intrathecal injection of anti-IL-6 neutralizing antibody alleviated these pain-related behaviors. Furthermore, IL-6 was reported to be intimately linked to nociceptive plasticity by enhancing translation in sensory neurons [42, 43]. IL-6 was also demonstrated to contribute to nociceptor sensitization and central sensitization [44–47]. These studies suggested an important role of IL-6 in pathological pain, indicating that the targeting of IL-6 or its receptor may reveal novel therapeutic interventions for the management of pathological pain. Moreover, humanized anti-IL-6R monoclonal antibody has exhibited excellent efficacy and safety against numerous diseases [48–50]. Therefore, here we review the current evidence of the role of IL-6 in the generation of pathological pain caused by bone cancer, peripheral nerve injury, spinal cord injury, chemotherapy-induced peripheral neuropathy, complete Freund’s adjuvant (CFA) injection, or carrageenan injection.

## IL-6 and cancer pain

Advanced prostate, lung, and breast cancer frequently metastasize to the bone, which causes 75–90 % of these patients to experience severe pain [51–53]. There is growing body of evidence demonstrating that IL-6 plays a vital role in various aspects of tumor behaviors including cell proliferation, migration, invasion, differentiation, and angiogenesis [54–57]. In this review, we focus on the critical role of IL-6 in pain caused by bone metastasis.

The involvement of IL-6 in BCP was first reported by Dong et al. [58], who used a rat model of BCP. In this study, the reverse transcription polymerase chain reaction (RT-PCR) results showed that the mRNA levels of IL-6 were considerably increased on 16 days after tumor cell implantation (TCI). Furthermore, intrathecal administration of EphB1-Fc significantly suppressed the mRNA levels of IL-6 in the spinal cord, indicating a downstream role of IL-6 in the analgesic effect of EphB1-Fc. In another study, immunohistochemistry and enzyme-linked immuno-sorbent assay (ELISA) work revealed that spinal IL-6 levels were significantly increased on day 12 after TCI [59]. It was found that propentofylline (PPF), a glial modulating agent, could alleviate pain hypersensitivity after TCI; in addition, the intrathecal injection of PPF markedly inhibited the expression of IL-6. Recently, it was shown that the intrathecal injection of tanshinone IIA, an ingredient in a traditional Chinese medicine, attenuated thermal hyperalgesia in a mouse model of BCP by inhibiting the release of pro-inflammatory cytokines [60]. More recently, Lu et al. [61] provided evidence for the persistent involvement of inflammation in the development of BCP; JWH-015, a selective cannabinoid receptor agonist, reduced the expression of pro-inflammatory cytokines in a time-dependent manner, thereby exerting an anti-nociceptive effect. Using conditional knockout mice lacking gp130 specifically in nociceptors, Andratsch et al. [62] uncovered that gp130 expressed in peripheral pain sensing neurons is critically required for the development of cancer pain. In addition, Quarta et al. [63] have shown the first genetic evidence that gp130 in Nav1.8 expressing primary afferents contributes to the maintenance of nociceptor sensitization in a mouse model of cancer pain. They found that mice with a null mutation of gp130 (gp130^−/−^) showed signs of nociceptor sensitization and hypersensitivity to mechanical stimuli in the early stage. However, gp130^−/−^ mice significantly recovered from hypersensitivity in the later stage, indicating that gp130 signal transducer plays a substantial role in regulating mechanical hypersensitivity particularly in the maintenance phase of cancer pain. The findings from the studies above implicate a role of IL-6 in the progression of cancer pain. However, the underlying mechanisms of IL-6 in the development of BCP were not investigated until Fang et al. [20]. Using RT-PCR and Western blotting, the expression levels of IL-6 and sIL-6R in the ipsilateral L4 and L5 DRG were found to be remarkably higher in BCP rats than in sham rats. Additionally, the intrathecal administration of FIL-6, a mixture of IL-6 and sIL-6R, induced hyperexcitability of nociceptive DRG neurons acutely isolated from naive rats and caused mechanical allodynia and thermal hyperalgesia in naive rats, suggesting that increased IL-6 contributed to the pathogenesis of BCP. Furthermore, both pretreatment and posttreatment with sgp130, a potent IL-6/sIL-6R trans-signaling inhibitor, remarkably attenuated the bone cancer induced overexcitability of DRG neurons and hyperalgesia in BCP rats, indicating that IL-6/sIL-6R trans-signaling was involved in the development of BCP by inducing DRG neurons hyperexcitability. More importantly, they found that transient receptor potential vanilloid channel type 1 (TRPV1) was the downstream target on which the enhanced expression of IL-6 in DRG neurons exerted its effects associated with the development of BCP. Activation of the JAK/PI3K signaling pathway was required for both the FIL-6-induced functional upregulation of TRPV1 in DRG neurons and pain hypersensitivity in naive rats. This study provided various lines of evidence for a novel intracellular pathway, the IL-6/JAK/PI3K/TRPV1 signaling cascade, which may underlie the development of peripheral sensitization and BCP.

## IL-6 and neuropathic pain

Neuropathic pain is a chronic pain condition caused by a primary lesion in or dysfunction of the nervous system and is characterized by spontaneous and evoked pain [64–66]. This type of pain is commonly observed in patients with cancer, diabetic peripheral neuropathy, herpes infection, spinal cord injury (SCI), or multiple sclerosis [67, 68]. Although there is no systematic classification system, neuropathic pain could be classified based on the etiology of the insult to the nervous system or the anatomical distribution of the pain [69]. Various animal models have been established to explore the mechanisms of neuropathic pain of different etiologies, including peripheral nerve injury, SCI, and chemotherapy-induced peripheral neuropathy. Using these animal models, a great deal of basic research has been performed to elucidate the mechanisms of neuropathic pain, which are complex and involve both peripheral and central pathophysiological phenomena. Following peripheral nerve injury, A-δ fiber and C-fiber primary afferent neurons become abnormally sensitive and develop pathological spontaneous activity, leading to peripheral sensitization [70]. This sensitization triggers the production of mediators, alteration of ion channels, and sprouting of nerves endings. These activities provoke secondary changes in central sensory processing, thereby contributing to spinal cord hyperexcitability and central sensitization [71]. Recently, converging lines of evidence have indicated that IL-6 plays a critical role in neuropathic pain caused by peripheral nerve injury, SCI, and chemotherapy-induced peripheral neuropathy.

## IL-6 and peripheral nerve injury

Most studies have used an animal model of peripheral nerve injury to explore the relationship between IL-6 and neuropathic pain. A growing body of research has demonstrated that IL-6 plays a role in the pathogenesis of neuropathic pain. The involvement of IL-6 in peripheral neuropathy was first found in a rat model of sciatic cryoneurolysis (SCN), in which the sciatic nerve was frozen to induce nerve injury [72]. The immunohistochemical data resulting from this model showed that IL-6-like immunoreactivity was significantly higher in both the dorsal and ventral horns in SCN rats than in those of normal rats. Furthermore, intrathecal administration of recombinant human IL-6 could mimic and even potentiate pain behavior after SCN. These results provided evidence that IL-6 may be involved in the development of neuropathic pain following SCN. In a subsequent study, they demonstrated that IL-6 mRNA was significantly elevated in both the dorsal and ventral horns in a neuropathic pain model of spinal nerve cryoneurolysis and spinal nerve tight ligation using in situ hybridization and a digoxigenin-labeled oligonucleotide [73]. In addition, they further demonstrated that an intrathecal injection of anti-IL-6 antibody could attenuate L5 spinal nerve transection-induced mechanical allodynia [74], providing further evidence for the role of central IL-6 in the etiology of mechanical allodynia following peripheral nerve injury. In another study, Ramera et al. [75] reported that spinal nerve lesion-induced mechanical allodynia was attenuated and delayed in IL-6 knockout mice, indicating a role of IL-6 in the initiation of neuropathic pain. Similar results were reported in IL-6^−/−^ mice using chronic constriction injury (CCI) model [76]. Using in situ hybridization, Brazda et al. [77] were the first group to show that IL-6 and IL-6R synthesis was increased in remote cervical DRG not associated with the nerve injury following CCI. They found that unilateral CCI induced the bilateral elevation of IL-6 and IL-6R mRNAs not only in L4–L5 DRG but also in remote cervical DRG, suggesting a general neuro-inflammatory reaction of the nervous system to local nerve injury. They further confirmed their hypothesis in a subsequent study [78].

The above studies demonstrated the participation of IL-6 in the pathogenesis of peripheral nerve injury-induced neuropathic pain. However, the underlying molecular and cellular mechanisms were not investigated. In an in vivo and in vitro study, Ma et al. [79] reported the involvement of prostaglandin E2 (PGE2) in the upregulated expression of IL-6 by invading macrophages following partial sciatic nerve ligation (PSNL). The immunostaining results of the in vivo study confirmed the dramatically increased number of IL-6-immunoreactive cells in the injured nerve of PSNL rats. The in vitro results showed that the levels of both PGE2 and IL-6 released from cultured cells derived from injured nerves were significantly increased, as well as that these elevated levels could be suppressed by non-selective and selective COX2 inhibitors. Interestingly, although PGE2 treatment did not remarkably increase the level of IL-6 released from cultured cells derived from uninjured nerve, it did increase the level of IL-6 released from injured nerve-derived cells in a concentration- and time-dependent manner. Moreover, both a selective PGE2 receptor 4 (EP4) antagonist (L-161982) and a protein kinase C (PKC) inhibitor (calphostin C) dramatically suppressed IL-6 release. These findings suggested that PGE2 was involved in mediating the upregulation of IL-6 occurring in invading macrophages via the EP4 receptor and the PKC pathway. In a subsequent study, they also demonstrated the role of PGE2 in the synthesis of IL-6 in primary sensory neurons following PSNL [80]. The in vivo data showed that injured nerve-derived PGE2 contributed to the de novo synthesis of IL-6 in damaged medium and large size DRG neurons following PSNL by activating the EP4 receptors. The in vitro data showed that EP4 receptor, PKA, PKC, ERK/MAPK, CREB, and NF-kB signaling pathways were involved in PGE2-induced IL-6 production in DRG neurons. These results provided evidence that facilitating the de novo synthesis of IL-6 in injured medium and large size DRG neurons was a new mechanism underlying the role of injured nerve-derived PGE2 in the development of neuropathic pain.

As IL-6 mainly activates the JAK/STAT transduction pathway, Dominguez et al. [81] investigated the possible activation of this signaling system in the spinal cord using an SNL model. It was shown that phospho-STAT3 (p-STAT3) in microglial cells of the spinal cord dorsal horn was significantly increased in SNL rats compared with sham rats and that inhibiting the STAT3 pathway attenuated both mechanical allodynia and thermal hyperalgesia in SNL rats. In line with previous studies, they found a massive induction of IL-6 mRNA expression in DRG and an increased concentration of IL-6 in the spinal cord dorsal horn. In addition, the intrathecal injection of anti-rat IL-6 antibodies prevented the SNL-induced accumulation of p-STAT3 in the spinal cord. Together, these data suggest that IL-6 plays a major role in the activation of the spinal JAK/STAT3 pathway after SNL and that this transduction pathway participates in the development of neuropathic pain.

It was reported that tumor necrosis factor-α (TNF-α), which binds to tumor necrosis factor receptor 1 (TNFR1) and induces NF-kB and p38 MAPK activation, was also upregulated following peripheral nerve injury [82, 83]. Therefore, Lee et al. [84] investigated whether TNFR1 regulates IL-6 expression through NF-kB or p38 MAPK activation in the spinal cord and DRG using a CCI model. They found that the CCI-induced upregulation of IL-6 expression was suppressed by intrathecal injection of a TNFR1 antisense oligonucleotide and an NF-kB decoy, but not by a p38 MAPK inhibitor, suggesting that TNFR1 induces IL-6 upregulation through NF- kB activation, but not p38 MAPK activation, in a CCI model. In a subsequent study, they further examined whether IL-6 regulates CX3CR1 expression through p38 MAPK activation in the spinal cord of CCI rats [21]. It was shown that CX3CR1 expression and p38 MAPK activation in the ipsilateral spinal dorsal horn were significantly increased following CCI and that an intrathecal injection of anti-IL-6 neutralizing antibody dramatically decreased both CX3CR1 expression and p38 MAPK activation. Additionally, naïve rats treated with exogenous recombinant rat IL-6 (rrIL-6) showed increased spinal CX3CR1 expression and p38 MAPK activation. Furthermore, treatment with a p38 MAPK-specific inhibitor, SB203580, suppressed the increase in CX3CR1 expression induced by either CCI or rrIL-6 treatment. These results indicated that IL-6 induces microglial CX3CR1 expression in the spinal cord after peripheral nerve injury through p38 MAPK activation, which demonstrates a new mechanism of neuropathic pain.

Several drugs have been reported to alleviate neuropathic pain, and this alleviation was accompanied by decreased serum level of IL-6 [85–87]. Recently, the clinical involvement of IL-6 in peripheral nerve injury-induced pain was also reported. Ohtori et al. [88] found that an epidural injection of an anti-IL-6R monoclonal antibody, tocilizumab, onto the spinal nerve alleviated radicular leg pain, numbness, and low back pain without causing adverse events in 60 patients with lumbar spinal stenosis-induced sciatica.

## IL-6 and spinal cord injury

Approximately 70 % of SCI patients have been reported to have chronic pain, and the pathogenesis of which remains largely unknown [89, 90]. Guptarak et al. [22] conducted a study to investigate the role of IL-6 in spinal cord injury pain (SCIP) using a clinically relevant rat contusion model. They found that only SCI rats that developed mechanical allodynia showed elevated IL-6 levels. Their immunocytochemistry results showed that increased IL-6 was predominantly co-localized with reactive astrocytes. Furthermore, one systemic injection of neutralizing IL-6 receptor antibody (IL-6R Ab) abolished the SCI-induced allodynia. As the humanized IL-6R Ab tocilizumab is approved by the Food and Drug Administration, they proposed that tocilizumab may become a novel and potentially effective means of managing SCIP. In another study, Murakami et al. [91] reported the beneficial effects of an anti-mouse IL-6R Ab (MR16-1) on neuropathic pain. The ELISA data showed that IL-6 levels between 24 and 72 h after SCI were significantly decreased in mice treated with MR16-1. Additionally, their behavioral data suggested that MR16-1 could alleviate hyperalgesia in SCI mice. The findings from these two studies indicate that IL-6/IL-6R trans-signaling may be a potential target for the treatment of SCIP.

## IL-6 and chemotherapy-induced peripheral neuropathy

Chemotherapy-induced peripheral neuropathy (CIPN) is a common consequence of several antineoplastic agents and can severely impact patients’ long-term quality of life [92, 93]. However, contradictory results have been reported, and the mechanisms of CIPN have remained unclear. In an in vivo study, three animal models of CIPN (i.e., rats treated with cisplatin or vincristine and mice treated with paclitaxel) were used to study the peripheral roles of IL-6 in painful CIPN [94]. This study first reported that IL-6 treatment could prevent the painful behavior of CIPN without interfering with the anti-tumor activity of these chemotherapeutic regimens, suggesting a potential neuroprotective effect of IL-6 on CIPN. In another study, the role IL-6 in vincristine-induced mechanical allodynia was examined using a mouse model of CIPN [95]. It was found that the expression of IL-6 was increased in CIPN mice and was co-localized with macrophage, as indicated by double immunostaining. Moreover, IL-6 neutralizing antibody considerably reduced vincristine-induced mechanical allodynia. In addition, the incidence of vincristine-induced mechanical allodynia was lower in IL-6 knockout mice than in wild-type mice. All of these results suggest that IL-6 plays a vital role in vincristine-induced mechanical allodynia. However, further investigation is required to determine whether IL-6, IL-6 neutralizing antibody or both can alleviate hyperalgesia and the associated underlying mechanisms. These conflicting results may be due to the different drug administration methods and animals used. Recently, a clinical study reported that IL-6 and sIL-6R levels were significantly higher after the conclusion of chemotherapy in breast cancer patients with CIPN than in those without CIPN, providing the first clinical evidence of the involvement of IL-6 in CIPN [96].

## IL-6 and inflammatory pain

Inflammatory pain is a common clinical symptom of inflammatory diseases and is characterized by hyperalgesia due to the sensitization of primary nociceptive neurons [97, 98]. Our previous study revealed that cannabinoid CB2 receptors (CB2Rs) are involved in the anti-nociceptive effect of electroacupuncture (EA) on inflammatory pain [99–101]. However, it was not clear how CB2R activation contributed to the anti-nociceptive effect of EA. Therefore, we conducted a study to investigate the effects of CB2R activation and EA on the expression level of several cytokines including IL-6 in a CFA rat model of inflammatory pain [24]. Using RT-PCR and Western blotting, we found that the mRNA and protein levels of IL-1β, IL-6, and TNF-α were significantly higher in CFA rats than in control rats. Moreover, treatment with EA or the selective CB2R agonist AM1241 significantly decreased the mRNA and protein levels of IL-1β, IL-6, and TNF-α in CFA rats. In addition, pretreatment with the specific CB2R antagonist AM630 significantly reversed the inhibitory effect of EA on IL-1β, IL-6, and TNF-α in CFA rats. These results suggested that EA suppressed the expression of IL-1β, IL-6, and TNF-α through CB2R activation, resulting in an analgesic effect. In another study, Sun et al. [102] found that tanshinone IIA attenuated the development of CFA-induced mechanical and thermal hypersensitivity, which was concomitant with downregulation of the spinal IL-6 level. Recently, Xu et al. [103] reported that triptolide, a traditional Chinese medicine ingredient, attenuated CFA-induced inflammatory pain by inhibiting spinal glia activation in rats. Pro-inflammatory cytokine levels were significantly increased after CFA injection. Furthermore, triptolide treatment reduced the levels of pro-inflammatory cytokines in the spinal cord. These results suggested that IL-6 may play a role in the pain-suppression effect of triptolide. More recently, Yang et al. [104] explored the possible mechanisms of the analgesic effect of oxysophocarpin, an alkaloid extracted from *Sophora alopecuroides*, on carrageenan induced inflammatory pain in mice. They found that IL-1β, IL-6, TNF-α, and PGE2 was significantly higher in mice with induced inflammatory pain than in sham mice and that the oxysophocarpin treatment markedly decreased their production. These findings demonstrated that IL-6 could potentially serve as a downstream target of several drugs to relieve inflammatory pain. However, it is still unclear how IL-6 suppression contributes to the alleviation of inflammatory pain. Therefore, further studies are needed to explore the molecular and cellular mechanisms of IL-6 in inflammatory pain. Our recent studies have shown that JAK2/STAT3 signaling may be involved.

## Conclusions

**Fig. 1:**
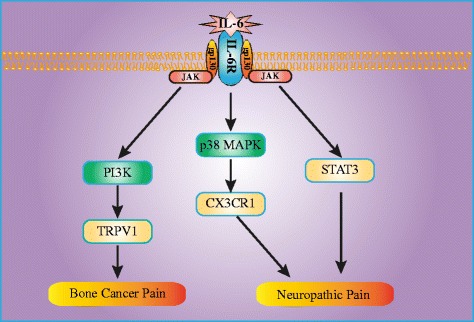
Schematic representation of the downstream mechanism of IL-6 in the processing of bone cancer pain and neuropathic pain. *IL-6* interleukin-6, *IL-6R* interleukin-6 receptor, *JAK* Janus-activated kinase, *PI3K* phosphoinositide 3-kinase, *TRPV1* transient receptor potential vanilloid channel type 1, *MAPK* mitogen-activated protein kinase, *STAT3* signal transducer activator of transcription 3

**Fig. 2:**
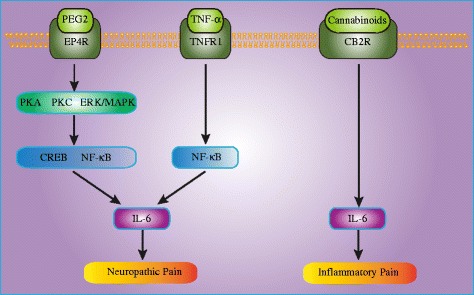
Schematic representation of the possible upstream mechanism of IL-6 in the processing of neuropathic pain and inflammatory pain. *PGE2* prostaglandin E2, *EP4R* prostaglandin E2 receptor 4, *PKA* protein kinase A, *PKC* protein kinase C, *ERK* extracellular signal-regulated kinase, *MAPK* mitogen-activated protein kinase, *CREB* cAMP-response element binding protein, *NF-kB* nuclear factor kappa B, *TNF-α* tumor necrosis factor-α, *TNFR1* tumor necrosis factor receptor 1, *IL-6* interleukin-6, *CB2R* cannabinoid CB2 receptor

By reviewing the current evidence, we discussed the relationship between IL-6 and pathological pain (Figs. 1 and 2). These studies provided robust evidence that IL-6 plays a critical role in the pathogenesis of BCP, neuropathic pain, and inflammatory pain. Treatment with anti-IL-6 or anti-IL-6R neutralizing antibody attenuates mechanical allodynia and thermal hyperalgesia caused by pathological pain, indicating that inhibitors of IL-6 or its receptors may be novel and beneficial therapeutic tools for pathological pain management. Although IL-6 plays vital roles in host defense and homeostasis maintenance, the overproduction of IL-6 causes the onset or development of several diseases. Therefore, novel therapeutic strategies using IL-6 or its receptors have been developed and successfully used for the treatment of numerous diseases. It was reported that tocilizumab, a humanized anti-IL-6R monoclonal antibody, improved the signs and symptoms of rheumatoid arthritis [105–110], juvenile idiopathic arthritis [111–113], and Castleman disease [114–116]. Furthermore, a recent prospective comparative cohort study provided evidence that single intradiscal injection of tocilizumab exerted a short-term analgesic effect in patients with discogenic low back pain [117]. Therefore, inhibitors of IL-6 or its receptors may be useful for the management of pathological pain. However, further research is warranted to extensively explore the exact role of IL-6 in pathological pain.

## Abbreviations

Akt, protein kinase B; BCP, bone cancer pain; CB2Rs, cannabinoid CB2 receptors; CCI, chronic constriction injury; CFA, complete Freund’s adjuvant; CIPN, chemotherapy-induced peripheral neuropathy; DRG, dorsal root ganglia; EA, electroacupuncture; ELISA, enzyme-linked immune-sorbent assay; EP4, PGE2 receptor 4; ERK, extracellular signal-regulated kinase; gp130^−/−^, null mutation of glycoprotein 130; IL-6, interleukin-6; IL-6R Ab, IL-6 receptor antibody; JAK, Janus-activated kinase; MAPK, mitogen-activated protein kinase; mIL-6R, membrane-bound IL-6 receptor; PI3K, phosphatidylinositol 3-kinase; PGE2, prostaglandin E2; PKC, protein kinase C; PPF, propentofylline; PSNL, partial sciatic nerve ligation; p-STAT3, phospho-signal transducer activator of transcription 3; rrIL-6, recombinant rat IL-6; RT-PCR, reverse transcription polymerase chain reaction; SCI, spinal cord injury; SCN, sciatic cryoneurolysis; sgp130, soluble form of gp130; sIL-6R, soluble form of IL-6R; TCI, tumor cell implantation; TNF-α, tumor necrosis factor-α; TNFR1, tumor necrosis factor receptor 1; TRPV1, transient receptor potential vanilloid channel type 1
